# Pedicled Cervical Lymphoadipose Tissue for Volume Reconstruction after Superficial Parotidectomy

**DOI:** 10.1155/2021/5574419

**Published:** 2021-05-07

**Authors:** Kyle P. Davis, Amy L. Fraser, Elizabeth O. Shay, Michael W. Sim

**Affiliations:** ^1^Indiana University School of Medicine, 340 West 10^th^ Street, Suite 6200, Indianapolis, IN 46202, USA; ^2^Department of Otolaryngology–Head and Neck Surgery, Indiana University School of Medicine, 1130 W. Michigan Street, Suite 400, Indianapolis, IN 46202, USA

## Abstract

Volume restoration is often required after parotidectomy due to the resultant facial contour deformity. Common procedures include local pedicled flaps, such as the sternocleidomastoid muscle flap, fat grafting, and even autologous free flaps, for more extensive defects. Local pedicled flaps have the advantage of a single surgical site, which spares the patient the added morbidity of a separate fat graft donor site, while simultaneously reducing the operative time. We report two cases of a novel reconstructive option using pedicled level I and II cervical lymphoadipose tissue for volume restoration after superficial parotidectomy. This reconstruction would be useful for patients with benign parotid lesions and inferior parotid defects. In addition, with maintained blood supply to this tissue, it would likely provide sustained bulk over time.

## 1. Introduction

The most common site for salivary gland tumors is the parotid gland, with surgical resection being the standard option in treatment [1]. A major drawback of these procedures is the resultant facial contour deformity, especially in the case of extensive resections. Some methods of restoring or minimizing facial contour defects involve adding bulk or volume from acellular dermis, autologous fat, or superficial muscular aponeurotic system flap [2]. To mitigate volume loss over time, larger defects require more vascularized flaps. Pedicled flaps, such as the sternocleidomastoid (SCM) muscle flap, supraclavicular artery flap, and submental flap, and for even larger defects, autologous free tissue flaps, may be required [2, 3]. Local pedicled flaps, however, can provide some advantages over utilization of free flaps.

It is well known that grafts exhibit volume loss over time, with both muscular and fatty tissue atrophy [2, 4], but Liang et al. found that using nonmuscular tissue, a pedicled submandibular gland flap, provided durable bulk and restored facial contour after parotidectomy. In addition, local pedicled flaps carry the advantage of a single surgical site, sparing the patient the added morbidity of a separate fat graft donor site, as well as reduced operative time and postoperative care compared to free flaps [2, 3]. We report two cases of utilizing a cervical lymphoadipose tissue pedicled flap that may have similar advantages for volume restitution following superficial parotidectomy for benign parotid lesions.

## 2. Case Presentation

### 2.1. Case 1

An 80-year-old Caucasian male presented to our clinic for evaluation and management of a right parotid tail mass that had been present for several years and had significantly increased in size over the past two months. Computed tomography (CT) revealed a 3.4 × 2.7 cm mass, and subsequent ultrasound guided core biopsy was consistent with a low-grade oncocytic neoplasm. On surgical evaluation, the tumor was found to be superficial to the facial nerve branches, so a superficial parotidectomy was performed with dissection and preservation of the facial nerve. Intraoperative frozen section analysis was performed confirming benign pathology, and final pathology would confirm an oncocytoma, measuring 4.8 × 3.7 by 2.3 cm.

After resection, there was a considerable mass defect that required volume reconstruction. Once frozen section pathology confirmed a benign tumor, the mass defect was reconstructed using a level I and II lymphofatty packet of tissue mobilized off the posterior aspect of the submandibular gland and SCM, advanced posteriorly and superiorly, and secured to the parotid fascia.

### 2.2. Case 2

A 58-year-old woman with a five-year history of a right-sided mass at the angle of the mandible sought evaluation after noting a recent increase in size with discoloration of the overlying skin. CT scan of the neck showed a mass measuring 3.2 × 2.9 cm involving the superficial lobe of the right parotid gland. Fine needle aspiration findings were consistent with a cystic lesion. On surgical evaluation, the tumor was found to be partially superficial and deep to the facial nerve branches. These were identified, dissected, and preserved while resecting the mass. Frozen section analysis demonstrated a papillary cystadenoma lymphomatosum tumor with cystic degeneration. Final pathology would confirm the frozen section diagnosis. The mass defect was reconstructed using a level I and II lymphofatty packet of tissue mobilized off the posterior aspect of the submandibular gland and SCM, advanced posteriorly and superiorly, and secured to the parotid fascia.

### 2.3. Surgical Technique

**Figure 1:**
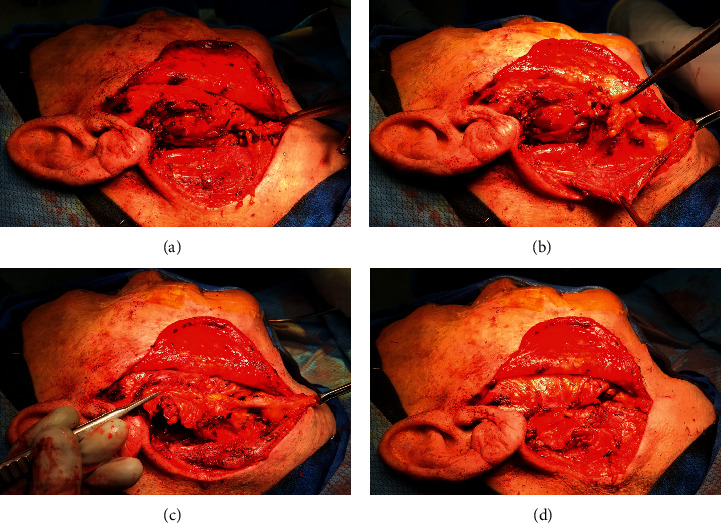
Volume reconstruction using pedicled lymphoadipose tissue after superficial parotidectomy. (a) Volume defect present after superficial parotidectomy. (b) Level I and II lymphoadipose tissue freed from surrounding structures with preservation of pedicle containing vascular supply. (c) Lymphoadipose tissue rotated posteriorly and superiorly into parotid defect. (d) Lymphoadipose tissue secured to parotid fascia.

**Figure 2:**
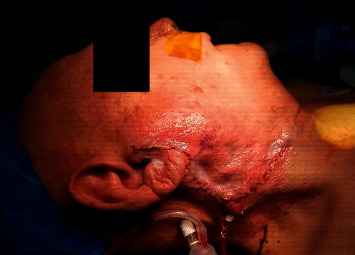
Wound closure after volume restoration.

**Figure 3:**
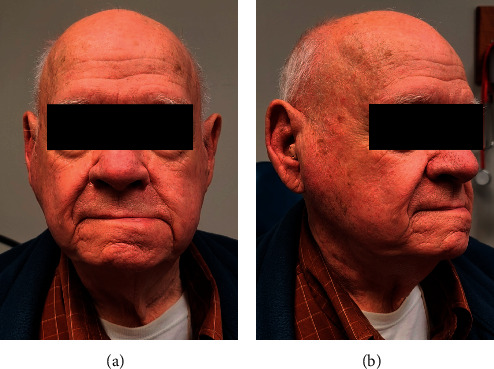
Five-month postoperative follow-up.

Surgical technique for volume reconstruction was performed similarly for both cases (Figure 1). The lymphoadipose tissue of levels I and II within the neck was mobilized from the mandible, marginal mandibular nerve, sternocleidomastoid muscle, spinal accessory nerve, and the submandibular gland. Vascular supply to this tissue was preserved from the occipital and facial vessels. This fibrofatty tissue was then rotated into the parotid defect and secured in place to the parotid fascia. With satisfactory volume restoration, the wound was then closed in a layered fashion (Figure 2). In essence, the surgical steps of elevating and mobilizing the tissue flap are very similar to the initial steps of a neck dissection in level II. On three- and five-month postoperative follow-up, the patients were noted to have durable volume reconstruction with excellent contour of the parotid region (Figure 3).

## 3. Discussion

After superficial or total parotidectomy, there is often a significant defect that requires volume reconstruction. There are several common locoregional options for relatively small defects including acellular dermis, sternocleidomastoid (SCM) muscle flap, and local fascia flaps [5]. Other reported regional options include both the submental flap [3] and submandibular gland (SMG) flap [2]. In addition, fat grafting is also frequently used but requires an additional operative site. As presented by this case series, pedicled cervical lymphoadipose tissue may be an additional surgical option to restore volume in patients who undergo parotidectomy for benign lesions. It is imperative to perform frozen section analysis prior to performing this reconstruction to greatly reduce the risk of transferring occult nodal metastatic disease from a malignant salivary gland tumor.

The use of pedicled cervical lymphoadipose tissues added minimal operative time at a single operative site and can be performed with no additional incisions. Furthermore, it has limited associated morbidity compared to SCM muscle flaps which can require harvesting of a considerable portion muscle. Skeletal muscle flaps also tend to atrophy over time which may lead to variability in results [2]. This reconstructive method involves maintenance of the tissue's vascular supply. The volume that remains long term is therefore more predictable, due to less atrophy, in comparison with free fat grafts that undergo considerable atrophy, the degree of which is hard to predict. Future studies, with longer follow-up time, would be useful to investigate the degree of volume that pedicled cervical lymphoadipose tissue maintains over time.

Potential limitations include variations in the amount of cervical lymphoadipose tissue and the location and size of the defect. The volume that can be replaced by this reconstructive method is dictated by patient anatomy and amount of tissue that is available. This limitation is mitigated by the advantage of this being a vascularized flap undergoing less atrophy in comparison with nonvascularized grafts as discussed above. Liang et al. found that vascular anatomy of the SMG flap prevented the coverage of the superoposterior aspect of the defect in some cases, which would also likely be the case when using pedicled lymphoadipose tissue. As in our cases, pedicled lymphoadipose tissue would be useful to cover inferior defects and could be used in conjunction with another reconstructive modality to provide optimal volume reconstruction. Again, this reconstructive technique should only be considered for benign parotid lesions, and we recommend frozen section analysis of the parotid tumor and any suspicious lymph nodes prior to reconstruction.

Continued utilization of this novel reconstructive option will allow for delineation comparison of long-term outcomes, complications, and patient satisfaction to more established reconstructive methods.

## Data Availability

No data were used to support this study.
